# Investigation of bedaquiline heteroresistance among *Mycobacterium tuberculosis* isolates from Pakistan

**DOI:** 10.1128/spectrum.02181-24

**Published:** 2025-02-24

**Authors:** Faiqa Rashid, Shaukat Iqbal, Sabira Tahseen, Yanlin Zhao

**Affiliations:** 1 Department of Bioinformatics and Biosciences, Capital University of Science & Technology, Islamabad, Pakistan; 2 National TB Control Program, National TB Reference Laboratory, Islamabad, Pakistan; 3 National Tuberculosis Reference Laboratory, Chinese Centre for Disease Control and Prevention, Beijing, China; ICON plc, London, United Kingdom

**Keywords:** bedaquiline, heteroresistance, mutational analysis

## Abstract

Bedaquiline is a key drug recommended by the WHO for the treatment of multidrug-resistant tuberculosis (MDR-TB) and extensively drug-resistant (XDR) TB, and its use could potentially shorten treatment duration with improved outcomes. However, resistance against this drug has increased, resulting in serious concerns. Heteroresistance is among the important obstacles to complicate the detection and treatment of MTB drug resistance. We investigated the presence of bedaquiline heteroresistant MTB isolates from Pakistan to highlight their relevance to bedaquiline resistance. Whole genome sequencing of 50 phenotypically bedaquline-resistant and 50 bedaquiline-sensitive isolates revealed 29% bedaquiline heteroresistance in our study. No significant association of patient variables (age, gender, region and history of anti-tuberculosis treatment [ATT]) was found, while drug resistance pattern among MDR + bedaquiline and XDR patterns (OR, 0.53 [0.01–0.26]; *P* ≤ 0.001 and OR, 0.09 [0.19–0.50]; *P* = 0.006) were significantly different to bedaquiline heteroresistance. Higher proportion of bedaquiline heteroresistant cases with no history of bedaquiline containing treatment was found. Most bedaquiline heteroresistant strains (*n* = 19) were from lineage 3, none of the strain bear mixed lineage, with *Rv0678* mutations (95%) being the most prevalent genetic marker. We identified both new mutations (n = 17) and reported mutations (n = 21) that contribute to bedaquiline heteroresistance.The strains with missense variants had the highest percentage of heteroresistance (56%). Bedaquiline heteroresistance is an important indicator of emerging bedaquiline resistance, predominantly observed in previously treated cases without mixed infections, suggesting a higher likelihood of acquired resistance. Our findings accentuate the complexity of bedaquiline heteroresistance and the need for better diagnostic and appropriate therapeutic treatment approaches for drug-resistant TB with bedaquiline-containing regimens.

**IMPORTANCE:**

This research is decisive as it investigates bedaquiline heteroresistance in *Mycobacterium tuberculosis* (MTB) isolates from Pakistan, the sixth highest burden country for drug-resistant tuberculosis (DRTB). Bedaquiline is a key drug in the treatment of MDR/XDR-TB, and the emergence of resistance to this drug threatens global efforts to control tuberculosis. Heteroresistance, where drug-susceptible and drug-resistant strains coexist, complicates detection and treatment, potentially leading to treatment failure. By focusing on MTB isolates from Pakistan, this study addresses a critical gap in understanding the prevalence and genetic mechanisms of bedaquiline resistance in a high-burden region. The use of whole genome sequencing (WGS) adds a cutting-edge approach to identifying mutations associated with resistance, offering valuable insights that could inform more effective treatment strategies and public health policies, ultimately contributing to the global fight against drug-resistant TB.

## INTRODUCTION

Tuberculosis (TB) is a primeval infectious disease and a global public health concern that is caused by *Mycobacterium tuberculosis* (MTB). Despite of the positive impacts of antimicrobial treatment of tuberculosis, it has also led to the widespread emergence of drug-resistant tuberculosis (DR-TB) that has become an distressing global problem (1). According to the World Health Organization (WHO), an estimated 13% (95% UI, 10%–19%) of all antimicrobial resistance-attributable deaths worldwide are caused by drug-resistant tuberculosis (DR-TB) (2). As stated in latest WHO Global TB report (2023), around 410,000 individuals developed MDR/RR-TB in the 2022, which is 3.9% (95% UI, 3.7%–4.1%) of the estimated incident TB cases (10.6 million) (3). The Southeast Asian region bears the highest DRTB burden (47%) region, while the Eastern Mediterranean and the American regions bear the least (3% each) (4).

Heteroresistance is the phenomenon of the coexistence of drug-susceptible and drug-resistant isolates (5). For instance, it turns complicated to detect MTB drug-resistant isolates due to heteroresistance that causes the dominance of drug-sensitive isolates over drug-resistant ones. It is the transitioning stage towards drug resistance (5) and is possibly caused by superinfection (infection with a resistant and susceptible strain simultaneously) or antibiotic selection pressure (a susceptible isolate converts to resistant because of any genomic mutation) (5, 6). In heteroresistance, bacteria can significantly utilize growth opportunities even in antibiotic environments, and this scenario makes the MTB isolates more vulnerable to drug resistance while undermining the treatment success (5). Previously published data indicated that the occurrence of heteroresistance in *Mycobacterium tuberculosis* isolates can range from 4.3% to 57% for various anti-tuberculosis drugs, including rifampicin (RIF), isoniazid (INH), fluoroquinolones (FQs), and pyrazinamide (PZA), whereas 83.9% heteroresistance was calculated for MDR (5–19).

Bedaquiline is a key drug recommended by the WHO for the treatment of MDR-TB and extensively drug-resistant (XDR-TB) (20–23) and is part of Bpal treatment regimen, which signifies it for DRTB treatment success in shorter duration (24). Bedaquiline is bactericidal in its action and executes its action while targeting mycobacterial ATP synthase (25, 26). Extensive and improper use of bedaquiline (BDQ) resulted in a rapid emergence of its resistance (27). The WHO has declared Pakistan as the sixth highest RR/MDR-TB burden country (4). In November 2015, bedaquiline was introduced in the treatment regimen of multidrug-resistant (MDR) and extensively drug-resistant (XDR) tuberculosis (TB) (28). A brief literature from Pakistan discussed the existence of BDQ resistance (29). There is a MTB heteroresistance study (30) for first-line drugs, but BDQ heteroresistance is not being highlighted ever. Moreover, there are some studies that described the phenomenon of bedaquiline heteroresistance in very few MTB isolates internationally (31–34). While acknowledging the worth of bedaquiline in DRTB treatment success, we designed the present study to investigate the presence of bedaquiline heteroresistance in MTB isolates from Pakistan. Using whole genome sequencing (WGS), we tried to highlight the Bdq heteroresistance-conferring mutations.

## MATERIALS AND METHODS

### Ethical approval and sample selection

**Fig 1:**
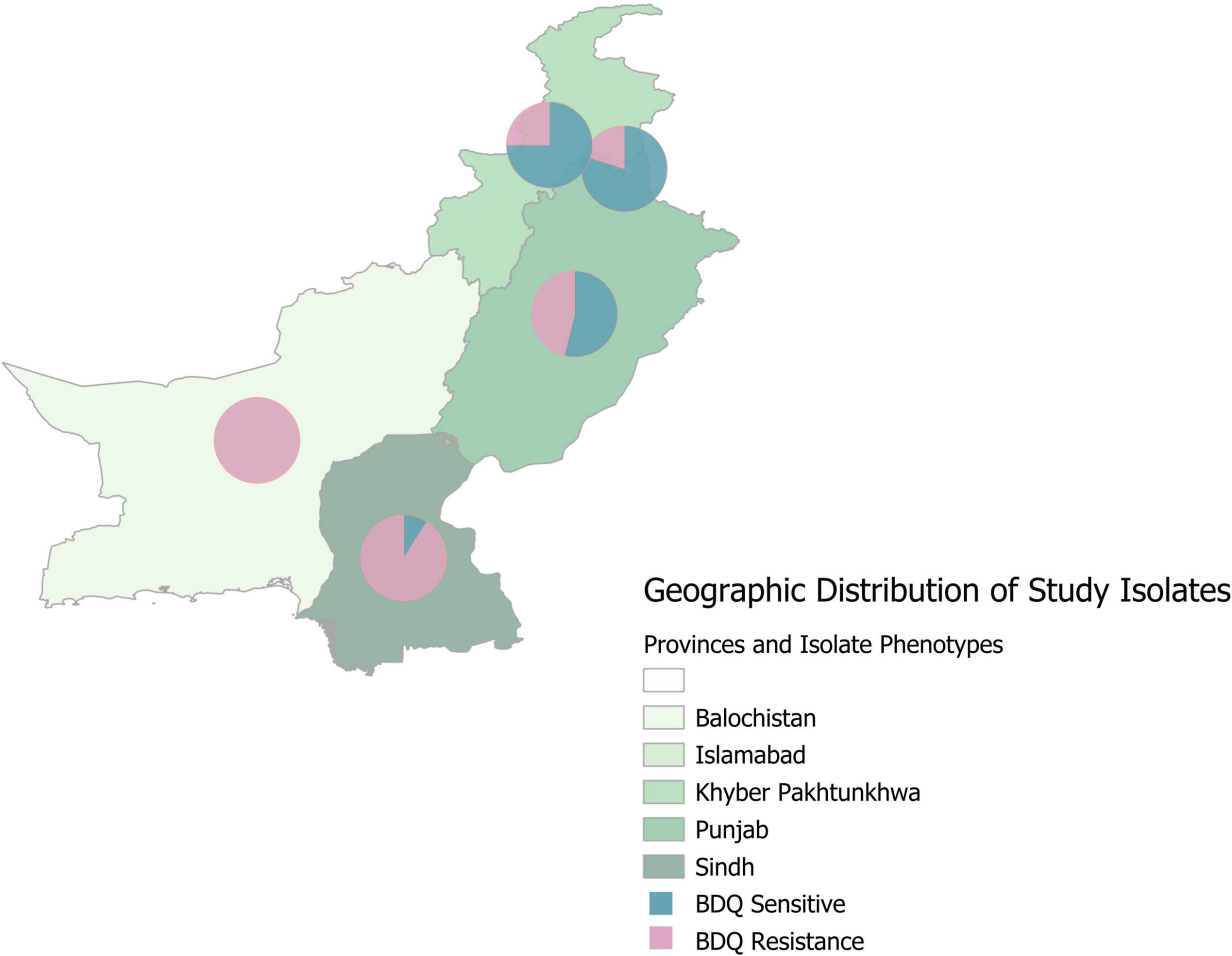
Geographic distribution of MTB study isolates from Pakistan. The map was created using Qgis 3.36.2.

The present study was approved by the ethical committee of the Capital University of Science & Technology, Islamabad, Pakistan. Culture isolates were selected from the archives of the National TB Reference Laboratory, Islamabad, Pakistan (NTRL), that originated from various regions of the country (Fig. 1), which were processed here from April 2022 to September 2023. For this study, four types of MTBC isolates were selected, including XDR-TB isolates reported to be resistant to BDQ and FQ, MDR-TB that is resistant to BDQ but not FQ, pre-XDR-TB, and RR/MDR-TB strains without any additional resistance to BDQ. From both groups, 50 bedaquiline-resistant cases were selected collectively. As a control group, an equal number of BDQ-sensitive cases were selected from the rifampicin (RIF)-resistant group.

### Sample processing

In NTRL, all samples were processed using the N-acetyl-L-cysteine–sodium hydroxide (NALC–NaOH) concentration method (35). All confirmed positive MTBC cultures were followed for drug susceptibility testing (DST) through the automated BACTEC MGIT 960 system (BD Diagnostic Systems, NJ, USA) (36–38).

### DNA extraction and whole genome sequencing

Genomic DNA from BDQ-resistant isolates was extracted by using the CTAB method (35). The extracted DNA was investigated for WGS after performing quality control steps. Sample sequencing was performed with the Illumina HiSeq2000 platforms.

### Bioinformatics analysis

FastQC was used to study sequence reads (www.bioinformatics.babraham.ac.uk/projects/fastqc/) as a preliminary data quality evaluation. Raw standard reads were omitted by utilizing trimmomatic software (36) and sequenced in comparison to the reference genome *H37Rv* through the BWA-mem alignment package (37). Single nucleotide polymorphism (SNP) call was made through the BCF/VCF software suite (38), followed by their FASTA format. While analyzing the WGS files of all heteroresistant isolates, the cutoffs of heteroresistance for the current study were defined between 5% and 95%, and the variants of quality ≥30 and depth ≥5 were annotated.

Evolutionary analysis of the selected strains was done by using IQ-Tree (39) that is an integrated tool for leading automatic and manual sequence alignment, inferring phylogenetic trees. Geographic distribution of study isolates was done using Qgis 3.36.2 Maidenhead (40). All the resulting mutations were searched in the mutation library of TBprofiler database (https://github.com/jodyphelan/tbdb).

### Statistical analysis

We analyzed the data of our current study by using IBM SPSS version 21. We applied univariate analysis with 95% CI to analyze the patient and bacterial potential factors for bedaquiline heteroresistance. Values were considered to be statistically significant at the significance level of 0.05. Heteroresistance percentage is indicated by the number of reads in the BWA-MEM pileup that contain mutations.

## RESULTS

Our study investigated the BDQ heteroresistance in 100 MTB strains, and among these isolates, 29 BDQ heteroresistance isolates were witnessed. We evaluated the potential patient and bacterial factors that could be associated to BDQ heteroresistance.

None of the patient variables (gender, age, region, previous history of anti-tuberculosis treatment) were statistically significant for bedaquiline heteroresistance. The proportion of BDQ heteroresistance in patients with a previous history of anti-tuberculosis treatment (*n* = 20) was higher (85%) than that in new patients (15%). MDR + BDQ R and XDR pattern tested through Phenotypic Drug Susceptibilty Testing (pDST) were the only bacterial variables that were computed to be statistically significant to BDQ heteroresistance (OR, 0.53 [0.01–0.26]; *P* ≤ 0.001 and OR, 0.09 [0.19–0.50]; *P* = 0.006). Majority of the study isolates belonged to lineage 3 (*n* = 73), likewise the highest number of isolates originated from lineage 3 (*n* = 19) (Table 1). These 19 cases comprise 16 BDQ-resistant and three BDQ-sensitive cases on phenotypic DST. None of the heteroresistant strain was detected with mix lineage.

**TABLE 1:** Potential markers for bedaquiline heteroresistance among MTB isolates^*a*^

Variable	Total no. of cases	No. of heteroresistance cases	Univariate analysis		
Patient			OR	95% CI	*P* value
Gender					
Male	57	14	1.20	0.38–3.78	0.74
Female	43	15			Ref
Age					
Young (≤25 years)	30	9	0.65	0.12–3.40	0.61
Adult (26–55 years)	46	15	0.77	0.17–3.37	0.73
Old (≥55 years)	24	5			Ref
Region					
ICT	5	0	–^*b*^	–	–
KPK	4	1	1.26	0.06–26.67	0.87
Balochistan	2	1	1.04	0.48–22.46	0.97
Sindh	11	3	5.18	0.84–31.63	0.07
Punjab	78	24			Ref
History of ATT					
New cases	80	12	1.15	0.33–3.97	0.82
Previously treated	20	17			Ref
Bacterial					
pDST					
Rif Mono	3	1	0.08	0.00–1.94	1.22
Rif R + BDQ R	1	0	–	–	–
MDR +BDQ R	19	11	0.53	0.01–0.26	<0.001*^c^
Pre-XDR	4	0	–	–	–
XDR	30	13	0.09	0.19–0.50	0.006*
MDR	43	4			Ref
MTB lineage					
Lineage 1	5	2	0.88	0.81–9.54	0.91
Lineage 2	7	4	0.31	0.03–2.73	0.29
Lineage 4	15	4	0.29	0.05–1.68	1.17
Lineage 3	73	19			Ref

^*a*^ ICT, Islamabad Capital Territory; KPK, Khyber Pakhtunkhwa; ATT, anti-tuberculosis treatment.

^*b*^ "-" signifies zero number of BDQ heteroresistance isolates in the respective group.

^*c*^ "*" indicates values which are significant at the level < 0.05.

**Fig 2:**
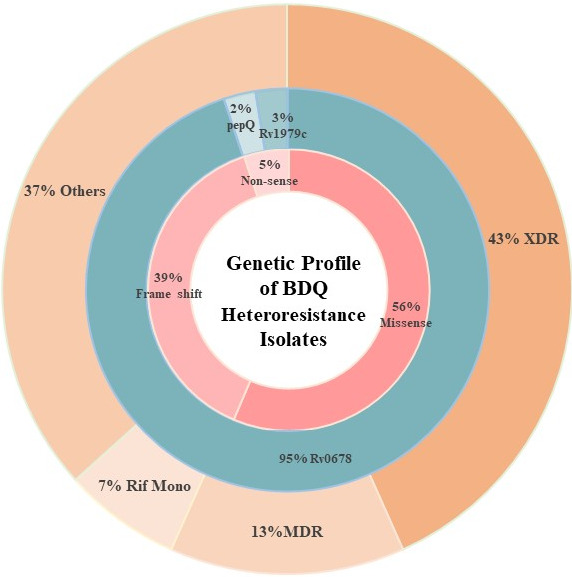
Genetic profile of BDQ-resistant isolates.

We represented the genetic profile of BDQ heteroresistance isolates in the form of a multilayer donut plot (Fig. 2). The outermost layer of the donut plot shows pDST patterns of BDQ heteroresistant isolates, with extensively drug-resistant strains being the most prevalent (43%), followed by Rif + BDQR and MDR + BDQR (37%), MDR (13%), and Rif Mono (7%). The middle layer highlights genetic markers associated with BDQ resistance, with mutations in *Rv0678* being the most common (95%), followed by *Rv1979c* (2%), and *pepQ* (3%). The innermost layer depicts mutation types, showing that 56% of heteroresistant strains had missense variants, 39% had frameshift variants, and 5% had nonsense variants.

The WGS of the 50 phenotypically BDQ-sensitive and 50 BDQ-resistant strains provided a variety of heteroresistance harboring mutations (Table 2). From all BDQ-resistant isolates, 24 BDQ-resistant and five BDQ-sensitive strains had heteroresistance mutations (Table 3), accounting for 29% heteroresistant population cumulatively. Mutations that existed in *Rv0678* (95%) include already reported (*n* = 20) as well as novel mutations (*n* = 16) (Table 2). We have only one mutation in the *Rv1979c* region (Phe300Leu) that was already reported, while a single unreported heteroresistance mutation was present in the *pepQ* gene of the MTB genome. The WGS data of current study isolates demonstrated that none of the strains were having heteroresistance mutation in *atpE* region (Table 3). The proportion of heteroresistance caused by each mutation was also computed. Among the reported BDQ resistance mutations, p.Ile67fs displayed heteroresistance phenomenon in maximal five BDQ-resistant strains, while majority of the novel mutations were present in a single isolate either from BDQ case or control group (Table 2). These BDQ heteroresistance-harboring mutations revealed different heteroresistance percentage from all three genetic markers ranging from 9% to 91%. According to WHO mutation catalog V2, we had four mutations (Asp47fs, Ile67fs, Leu32fs, and Glu49fs) that were reported to be associated with BDQ resistance. These four mutations were also present in non-heteroresistant strains (Table 4). Non-heteroresistant strains were also analyzed and evaluated for mutational analysis. A higher proportion of mutations were found in the *Rv0678* gene (*n* = 21), followed by *Rv1979c* (*n* = 5), *atpE* (*n* = 1), and *pepQ* (*n* = 1). The most common mutation in *Rv0678* was Asp47fs, identified in two BDQ-resistant strains (lineage 3). *Rv0678* mutations included nonsense, frameshift, and stop-gained types. A single nonsense mutation (Arg7Gln) occurred in the *pepQ* gene in four BDQ-sensitive strains. The *atpE* gene had one mutation (Ala63Pro) in a BDQ-resistant strain. In *Rv1979c*, six nonsense mutations were identified, with the most prevalent (Asp286Gly) found in two BDQ-resistant and two BDQ-sensitive strains. Other mutations in *Rv1979c* included Glu38Lys (*n* = 2), Met245Leu (*n* = 1), and Ser175Leu (*n* = 1). (Table 4)

**TABLE 2:** Mutational analysis of BDQ heteroresistance^*a*^

Gene	Mutation	No. of isolates	TB profiler	Source	Confidence with BDQ R
*Rv0678*	Arg34 Gln	1	Unreported	─	─
*Rv0678*	Asp88fs	1	Unreported	─	─
	Ile108fs	1	Unreported	─	─
*Rv0678*	Asp47fs	1	Reported	WHO catalog v2	Assoc with R
	Arg89Leu	1	Reported	WHO catalog v2	Uncertain significance
*Rv0678*	Gly87fs	1	Unreported	─	─
	Pro129fs	1	Reported	WHO catalog v2	Assoc w R - Interim
*Rv0678*	Arg72Trp	1	Reported	WHO catalog v2	Uncertain significance
*Rv0678*	Glu55*	1	Unreported	─	─
*Rv0678*	Ile67fs	5	Reported	WHO catalog v2	Assoc with R
*Rv0678*					
*Rv0678*					
*Rv0678*					
*Rv0678*					
*Rv0678*	Leu142Arg	1	Reported	WHO catalog v2	Uncertain significance
*Rv0678*	Leu32fs	2	Reported	WHO catalog v2	Assoc with R
*Rv0678*					
*Rv0678*	Ala124fs	2	Unreported	─	─
*Rv0678*					
*Rv0678*	Met10Lys	1	Unreported	─	─
*Rv0678*	Arg94Trp	1	Reported	WHO catalog v2	Uncertain significance
*Rv0678*	Phe19Ser	1	Reported	WHO catalog v2	Uncertain significance
*Rv0678*	Asn70Asp	1	Reported	WHO catalog v2	Assoc w R - Interim
*Rv0678*	Thr58fs	1	Unreported	─	─
*Rv0678*	Arg50Trp	1	Reported	WHO catalog v2	Uncertain significance
	Gln115fs	1	Unreported	─	─
*Rv0678*	Leu43Val	1	Unreported	─	─
*Rv0678*	Arg34Gln	1	Reported	WHO catalog v2	Uncertain significance
	Leu35Phe	1	Unreported	─	─
*Rv0678*	Tyr26Asp	1	Reported	WHO catalog v2	Uncertain significance
*Rv0678*	Glu49fs	1	Reported	WHO catalog v2	Assoc with R
*Rv0678*	p.Glu55fs	1	Reported	WHO catalog v2	Assoc w R - Interim
*Rv0678*	Gln51Glu	1	Unreported	─	─
*Rv0678*	Leu83Pro	1	Reported	WHO catalog v2	Uncertain significance
	Ala110fs	1	Reported	WHO catalog v2	Assoc w R - Interim
*Rv0678*	Leu83Phe	1	Unreported	─	─
*Rv0678*	Tyr145*	1	Reported	WHO catalog v2	Assoc w R - Interim
*Rv0678*	Gly78Trp	1	Reported	WHO catalog v2	Uncertain significance
	Ala86Val	1	Reported	WHO catalog v2	Uncertain significance
	Ala124fs	2	Unreported	─	─
	Gly65Val	1	Unreported	─	─
	Arg96Gln	1	Unreported	─	─
*Rv1979c*	Phe300Leu	1	Reported	WHO catalog v2	Uncertain significance
*pepQ*	Arg160Ser	1	Unreported	─	─

^*a*^ "─" signifies "Not Available".

**TABLE 3:** Heteroresistance strain^*a*^

Study #	BDQ pDST profile	Gene	Mutation	Heteroresistance (%)
BDQ_FR_004	BDQ-R	*Rv0678*	Arg34 Gln	11%
BDQ_FR_005	BDQ-R	*Rv0678*	Asp88fs	21%
			Ile108fs	44%
BDQ_FR_006	BDQ-R	*Rv0678*	Asp47fs	44%
			Arg89Leu	49%
BDQ_FR_009	BDQ-R	*Rv0678*	Gly87fs	57%
			Pro129fs	39%
BDQ_FR_010	BDQ-R	*Rv0678*	Arg72Trp	20%
BDQ_FR_011	BDQ-R	*Rv0678*	Glu55*	22%
	BDQ-R		Ile67fs	11%
	BDQ-R		Leu142Arg	26%
BDQ_FR_017	BDQ-R	*Rv0678*	Leu32fs	28%
BDQ_FR_018	BDQ-R	*Rv0678*	Ala124fs	24%
BDQ_FR_020	BDQ-R	*Rv0678*	Met10Lys	31%
BDQ_FR_023	BDQ-R	*Rv0678*	Ala124fs	51%
	BDQ-R	*Rv0678*		55%
BDQ_FR_024	BDQ-R	*Rv0678*	Phe19Ser	23%
	BDQ-R	*Rv0678*		41%
BDQ_FR_025	BDQ-R	*Rv0678*	Thr58fs	15%
BDQ_FR_027	BDQ-R	*Rv0678*	Arg50Trp	16%
				29%
BDQ_FR_029	BDQ-R	*Rv0678*	Leu43Val	33%
BDQ_FR_030	BDQ-R	*Rv0678*	Arg34Gln	55%
				20%
BDQ_FR_031	BDQ-R	*Rv0678*	Ile67fs	9%
BDQ_FR_033	BDQ-R	*Rv0678*	Tyr26Asp	13%
	BDQ-R	*Rv0678*		18%
BDQ_FR_034	BDQ-R	*Rv0678*	Leu32fs	27%
	BDQ-R	*Rv1979c*	Phe300Leu	28%
BDQ_FR_036	BDQ-R	*Rv0678*	p.Glu55fs	74%
BDQ_FR_039	BDQ-R	*Rv0678*	Gln51Glu	20%
BDQ_FR_059	BDQ-S	*Rv0678*	Gly78Trp	10%
	BDQ-S		Ala86Val	9%
BDQ_FR_060	BDQ-S	*Rv0678*	Ala124fs	9%
BDQ_FR_066	BDQ-S	*Rv0678*	Gly65Val	67%
	BDQ-S	*pepQ*	Arg160Ser	9%
BDQ_FR_079	BDQ-R	*Rv0678*	Ile67fs	91%
	BDQ-R		Leu83Pro	11%
			Ala110fs	40%
BDQ_FR_080	BDQ-R	*Rv0678*	Ile67fs	81%
BDQ_FR_086	BDQ-R	*Rv0678*	Leu83Phe	70%
	BDQ-R	*Rv0678*	Tyr145*	12%
BDQ_FR_087	BDQ-R	*Rv0678*	Ile67fs	11%
BDQ_FR_091	BDQ-S	*Rv0678*	Arg96Gln	15%
BDQ_FR_099	BDQ-S	*Rv0678*	Ala124fs	34%

^*a*^ "*" signifies a non-sense mutation which resulted in the replacement of respective codon with a stop codon indicating the premature termination of protein. "#" signifies "Number".

**TABLE 4:** Mutational analysis of non-heteroresistance strains^*a*^

BDQ status	Strain name	Nucleotide position	Amino acid change	Gene	Lineage
BDQ R	BDQ_FR_001	139dupG	Asp47fs	*Rv0678*	3
BDQ R	BDQ_FR_012	417G > T	Met139Ile	*Rv0678*	3
BDQ R	BDQ_FR_013	493dupG	Asp165fs	*Rv0678*	3
BDQ R	BDQ_FR_016	198dupG	Ile67fs	*Rv0678*	4
BDQ R	BDQ_FR_002	139dupG	Asp47fs	*Rv0678*	3
BDQ R	BDQ_FR_021	209delA	Asn70fs	*Rv0678*	3
BDQ R	BDQ_FR_022	144dupC	Glu49fs	*Rv0678*	3
BDQ R	BDQ_FR_026	143C > T	Pro48Leu	*Rv0678*	3
BDQ R	BDQ_FR_003	140dupA	Asp47fs	*Rv0678*	3
BDQ R	BDQ_FR_035	296C > A	Ala99Asp	*Rv0678*	4
BDQ R	BDQ_FR_037	417G > T	Met139Ile	*Rv0678*	3
BDQ R	BDQ_FR_038	151C > G	Gln51Glu	*Rv0678*	4
BDQ S	BDQ_FR_041	426C > G	Asn142Lys	*Rv0678*	2
BDQ S	BDQ_FR_047	370G > C	Ala124Pro	*Rv0678*	3
BDQ S	BDQ_FR_067	259G > C	Gly87Arg	*Rv0678*	1
BDQ S	BDQ_FR_070	23A > G	Asp8Gly	*Rv0678*	3
BDQ R	BDQ_FR_008	201dupC	Ser68fs	*Rv0678*	3
BDQ R	BDQ_FR_081	144dupC	Glu49fs	*Rv0678*	3
BDQ R	BDQ_FR_083	18_19delGG	Val7fs	*Rv0678*	3
BDQ R	BDQ_FR_084	358G > A	Val120Met	*Rv0678*	3
BDQ R	BDQ_FR_089	439G > T	Glu147*	*Rv0678*	3
BDQ S	BDQ_FR_097	95T > C	Leu32Ser	*Rv0678*	2
BDQ S	BDQ_FR_004	20G > A	Arg7Gln	*pepQ*	3
BDQ S	BDQ_FR_030	20G > A	Arg7Gln	*pepQ*	3
BDQ S	BDQ_FR_049	20G > A	Arg7Gln	*pepQ*	3
BDQ S	BDQ_FR_062	20G > A	Arg7Gln	*pepQ*	3
BDQ R	BDQ_FR_032	187G > C	Ala63Pro	*atpE*	4
BDQ R	BDQ_FR_005	857A > G	Asp286Gly	*Rv1979c*	1
BDQ S	BDQ_FR_069	857A > G	Asp286Gly	*Rv1979c*	1
BDQ S	BDQ_FR_072	857A > G	Asp286Gly	*Rv1979c*	1
BDQ R	BDQ_FR_028	857A > G	Asp286Gly	*Rv1979c*	4
BDQ R	BDQ_FR_088	857A > G	Asp286Gly	*Rv1979c*	4
BDQ R	BDQ_FR_001	733A > C	Met245Leu	*Rv1979c*	3
BDQ R	BDQ_FR_005	524C > T	Ser175Leu	*Rv1979c*	1
BDQ R	BDQ_FR_031	112G > A	Glu38Lys	*Rv1979c*	3
BDQ S	BDQ_FR_042	112G > A	Glu38Lys	*Rv1979c*	3

^*a*^ "*" signifies a non-sense mutation which resulted in the replacement of respective codon with a stop codon indicating the premature termination of protein.

## DISCUSSION

The rising incidence of DRTB is the major hindrance in attaining the global goal of TB control. To improve the understanding of DRTB mechanics, it is decisive to unravel the demographic factors in addition to treatment history that contribute to the resistance of MTB strains against the anti-tuberculosis drugs (41). Resistance against any particular drug occurs due to mutation in the drug targets, mixed strain infection involving different MTB strains, and heteroresistance in drug target (26, 42). Previous studies deciphered the heteroresistance in various DRTB drugs, including bedaquiline, but there is an inadequate literature for the mutational analysis of BDQ heteroresistance that is being addressed in the current study.

The findings of the present study indicated 29% BDQ heteroresistance in our population of interest. Previously, in two relevant studies, the percentage of BDQ heteroresistance was computed as 21.01% (26) and 60% (32). The majority of the patients suffering from heteroresistance infection were in the adult age group in the current study, which is considered as the most productive age group (43). The higher proportion of this age group cannot be ignored as it acts as an important potential factor for heteroresistance in our results. Our data represent 20 patients with previous treatment, out of which 17 cases were presented as heteroresistant. We found four of these cases were previously cured with BDQ in their treatment regimen, and the rest of the cases only had a previous history of first-line ATT. Although history of ATT was not significantly associated to BDQ heteroresistance, higher proportion of previously treated heteroresistant cases with their previous treatment regimen and treatment outcome information can be useful to stratify the respective strains whether they are suffering from primary or acquired resistance. Our findings suggest the concentration of BDQ heteroresistance in previously treated cases with the possible occurrence of acquired resistance. Current research findings were somehow a little different than another study in that mix infection and heteroresistance among DRTB isolates were more prevalent in the young age group, which is indeed a growing age (44). The number of XDR in addition to MDR + BDQ-resistant strains was statistically significant in relation to the presence of heteroresistance, which represents the significance of bedaquiline in MDR and XDR patients’ treatment. Though the MDR group is the largest group in our study, there are very few number of heteroresistance cases present in this group (0.09%). Punjab is the most populated province in Pakistan (43) and where the maximum number of heteroresistance patients is found; concurrently, it was noticed that a high proportion of these heteroresistant cases were suffering from MDR-TB. These results indicate MDR is highly prevalent in Punjab as described in another study (43), and MDR patients who take BDQ as an integral part of their treatment are more prone to BDQ resistance because they are BDQ heteroresistant, which means half way to resistance. This scenario is another evidence that signifies the participation of BDQ heteroresistance in BDQ resistance occurrence.

The phylogeny of *M. tuberculosis* comprises four major lineages (L1–L4), each containing discrete strain types that may vary in their transmission action, resulting in severe disease manifestation (45). Lineage 3 was the most frequent lineage present in our data set, particularly in BDQ-heteroresistant isolates. Lineage 3 is the Central Asian lineage. A study from Pakistan on representation of drug-resistant MTB mutations and its transmission concluded with the same results in that 74.2% strains belonged to lineage 3 (46). Another investigation from Karachi, Pakistan, also represented lineage 3 as the most prevalent lineage (29). The maximum prevalence of lineage 3 in our Pakistani isolates reveals the molecular epidemiology of MTB in Pakistan (47).Many other studies also nominated Central Asian (CAS) as the most common MTB lineage (30, 48–52). A study from South Africa that focused on bedaquiline heteroresistance presented Beijing as the most predominant lineage (32). As mentioned earlier, in heteroresistant cases the susceptible MTB stains mask the resistant strains (5), so we can see that many of the BDQ-sensitive strains in the present research was proved phenotypically BDQ sensitive but heteroresistant on WGS analysis. There was no heteroresistant strain detected with mix lineage that increases the possibility of selection (acquired) resistance in these strains based on WGS, and this observation was also evident from the above-mentioned high proportion of BDQ-heteroresistant cases with no history of BDQ-containing treatment. Bedaquiline is a core drug used in the treatment of rifampicin-resistant tuberculosis. Several candidate BDQ resistance genes have been identified, but only a few genomic variants *atpE, pepQ, Rv1979c*, and *Rv0678* have been statistically linked to bedaquiline resistance (53). Our results provided us the mutation data in *Rv0678*, *pepQ*, and *Rv1979c* for bedaquiline-heteroresistant MTB species, but those mutations were not enough to be computed for statistical analysis as the majority of these mutations were harbored by a single strain. With relevance to our results, there was some evidence of BDQ heteroresistance reported only in *Rv0678* (32), but none of the study presented heteroresistance caused by mutations in *pepQ, atpE*, and *Rv1979c* gene targets. A study in 2023 used published phenotype data for BDQ resistance variants in *Rv0678, atpE*, *pepQ*, and *Rv1979c* genes in 756 *M. tuberculosis* isolates and applied Bayesian methods to estimate the posterior probability of bedaquiline resistance and concluded that the role of *Rv0678* and *atpE* is evident in BDQ resistance, but the role of *pepQ* and *Rv1979c* variants is uncertain. There was a high level of *in vitro* BDQ resistance observed for *atpE* as it can cause a loss of binding affinity of BDQ with its hotspot (54), but there was not a definite evidence of its involvement for clinical isolates (55). However, the accelerated expression of the MmpS5/MmpL5 efflux system resulted by the mutations in *Rv0678* gene is the main cause of clinical resistance to BDQ. Finally, there was a poor evidence of BDQ resistance in the *pepQ* and *Rv1979c* genes because of mutations, but still they exist as the biomarker of BDQ resistance (56). In a recent systematic review, the linkage between phenotypic BDQ-resistant and genomic variants in *atpE*, *Rv0678, Rv1979c*, and *pepQ* was analyzed (57), but only 0.006% (2/313) variants were statistically significant to BDQ resistance (58). According to WHO mutation catalog 2021, no mutations fulfilled the criteria for BDQ resistance (59), while the recent mutation catalog published in 2023 reported six mutations fulfilled this criteria of association with bedaquiline resistance (60). Therefore, the amount of evidence on BDQ genotype–phenotype association failed to affirm the use of gDST, but the data from WGS and pDST results added in the second addition of WHO mutation catalog somehow signified these techniques as a better mode for patient care. The variant type of our study isolates were also stratified, and missense mutation exceeds their involvement for BDQ heteroresistance in contrast to frame shift and non-sense mutations. Literature review provided the evidence that there was a low probability for synonymous mutations in *Rv0678* (3.3%), high (55.1%) for nonsense mutations in *Rv0678*, comparatively low for missense (31.5%) mutations and frameshift (30.0%) in *Rv0678*, and low for missense mutations in *pepQ* (2.6%) and *Rv1979c* (2.9%) (56).

The current BDQ heteroresistance study involved the mutational analysis of our BDQ case control isolates. We screened the WGS data to highlight all the mutations that were part of BDQ target genes and underwent mutations in these regions from our data set. As mentioned in Table 2, maximum mutations causing the heteroresistance phenomenon were located in the *Rv0678* region. Among the reported mutations, Asp47fs existed in a single isolate but possesses its significance in BDQ resistance and is being discussed in a variety of DRTB studies previously (32, 61–63). This mutation was proved to be involved in both primary as well as acquired BDQ resistance in addition to cross-resistance to clofazimine and poor treatment outcome (64). Another mutation Arg89Leu was studied recently in an epidemiological study of MTB from Georgia and was present with a frequency of 99.1%, while in our data set, it presented its heteroresistance with 49% (65). Ile67fs as one of most ancient and commonly occurring mutational marker existed in five stains with a heteroresistance level of 9%–91% (66), Various other mutations Arg72Trp, Leu142Arg, Arg94Trp, Phe19Ser, Arg50Trp, Glu49fs, Ala86Val, and Arg96Gln were part of our MTB strains, but these mutations confer BDQ resistance in the *Rv0678* region as reported in past experiments in other countries (53, 67–70). The presence of these mutations in relation to BDQ heteroresistance in our population emphasizes their upcoming fixation probability as confirmed BDQ-conferring mutations in Pakistani population enduring bedaquiline as added treatment. All the novel mutations discovered in this study were present in single isolates for all three markers for bedaquiline resistance (*Rv0678, Rv1979c*, and *pepQ*) with a varied percentage of heteroresistance and need to be further investigated in diverse and larger DRTB population for their fixation probability. The mutations found in non-heteroresistant isolates as well as in heteroresistant strains are providing the significance that, on acquiring possible true resistance in near future, these mutations would harbor as true resistant biomarkers. As a core part of DRTB treatment especially Bpal (bedaquiline, pretomanid, and linezolid), BDQ can improve the treatment outcomes for drug-resistant TB, but challenges in predicting BDQ heteroresistance and optimizing its use remain areas of concern. Our findings highlight the complexity of BDQ heteroresistance and the need for enhanced diagnostic and treatment strategies to manage drug-resistant TB effectively. Studies with larger sample size and regions with diverse genetic backgrounds and epidemiological patterns of TB might provide us more significant information about BDQ heteroresistance with the integration of multiple diagnostic approaches. The study primarily focused on the genetic and molecular aspects of BDQ heteroresistance based on phenotypic DST and WGS, with no MIC testing and limited clinical correlation regarding patient outcomes and treatment efficacy. Further research should aim to link genetic findings with clinical data.

## Data Availability

The FASTQ files for all 100 strains are uploaded on public repository and can be accessed at BioProject no. 1195559.
